# Supplementary material for: Identifying mutations in sd1, Pi54 and Pi-ta, and positively selected genes of TN1, the first semidwarf rice in Green Revolution
Source: Bot Stud. 2022 Mar 26;63:9. doi: 10.1186/s40529-022-00336-x (PMC8960516; doi:10.1186/s40529-022-00336-x)
Supplement: Supplementary file 1 — Additional file 1. TN1 Botanical studies. [file 40529_2022_336_MOESM1_ESM.docx]

1. **SUPPLEMENTARY FIGURES**


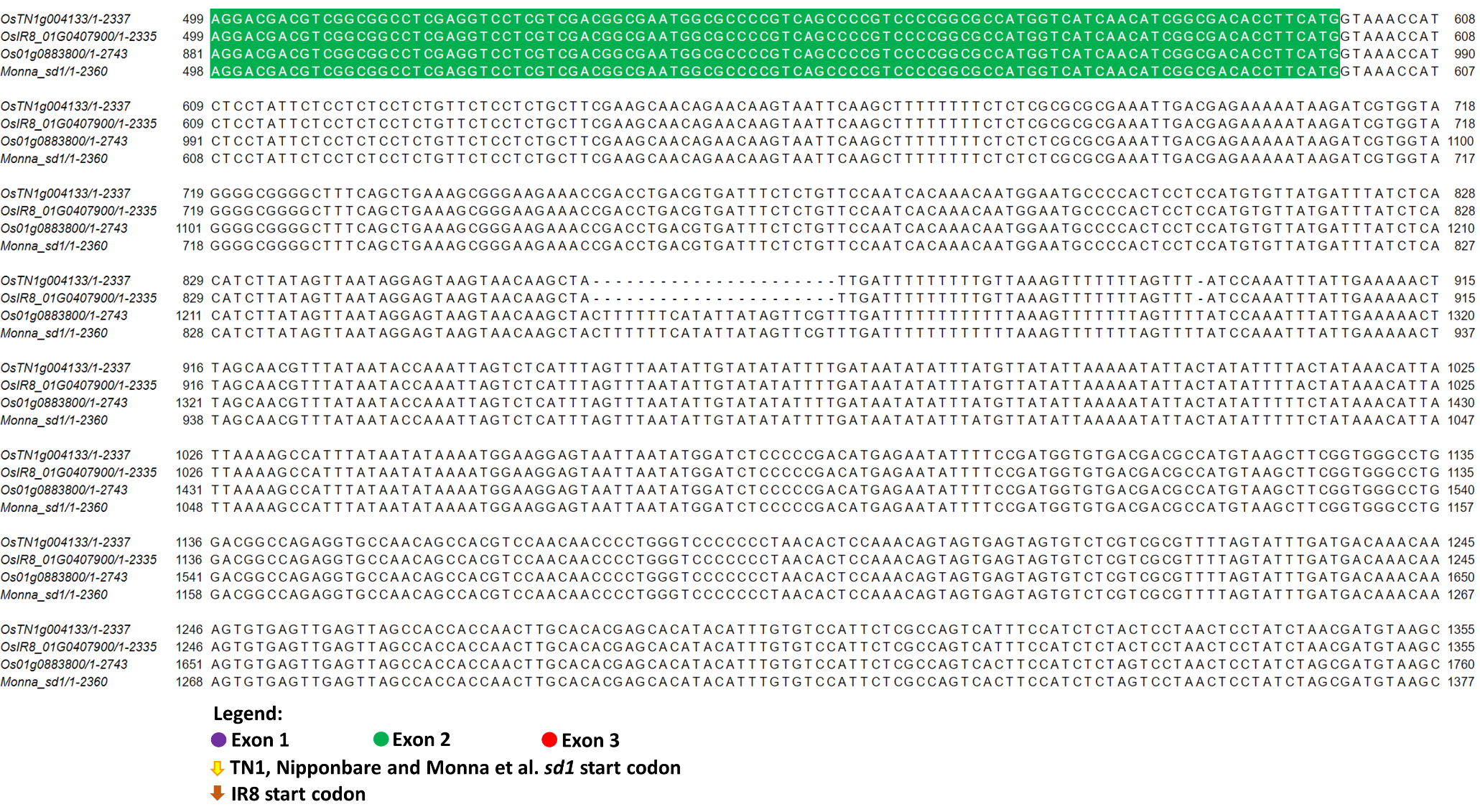


**Figure S1. Continuation of the *sd*1 exon alignment in Figure 1**. The sequence covering the first base of exon 1, up to the last base of exon 3 were used in the alignment. Monna et al.’s *sd*1 sequence was copied from its publication. The alignment continues in the next image.


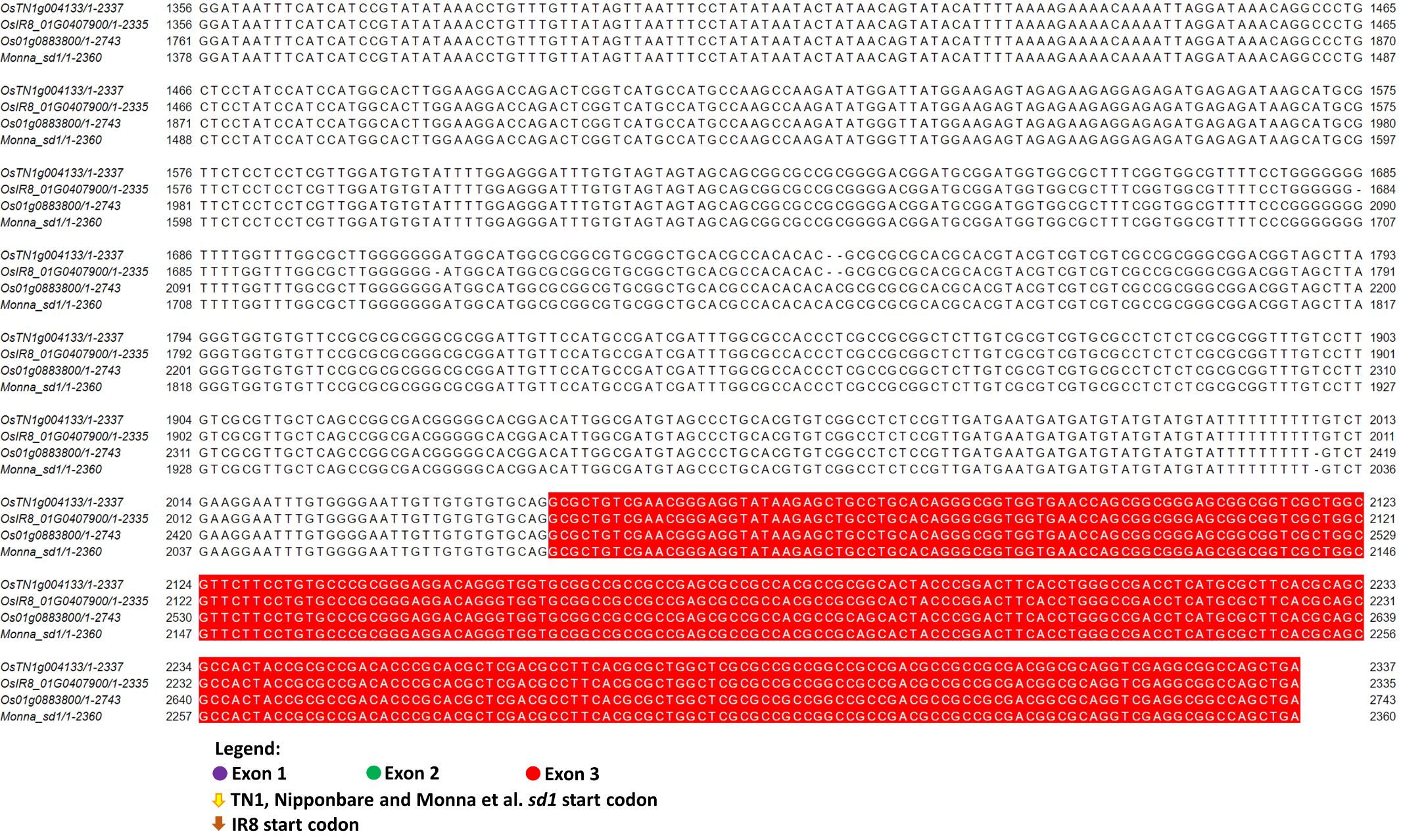


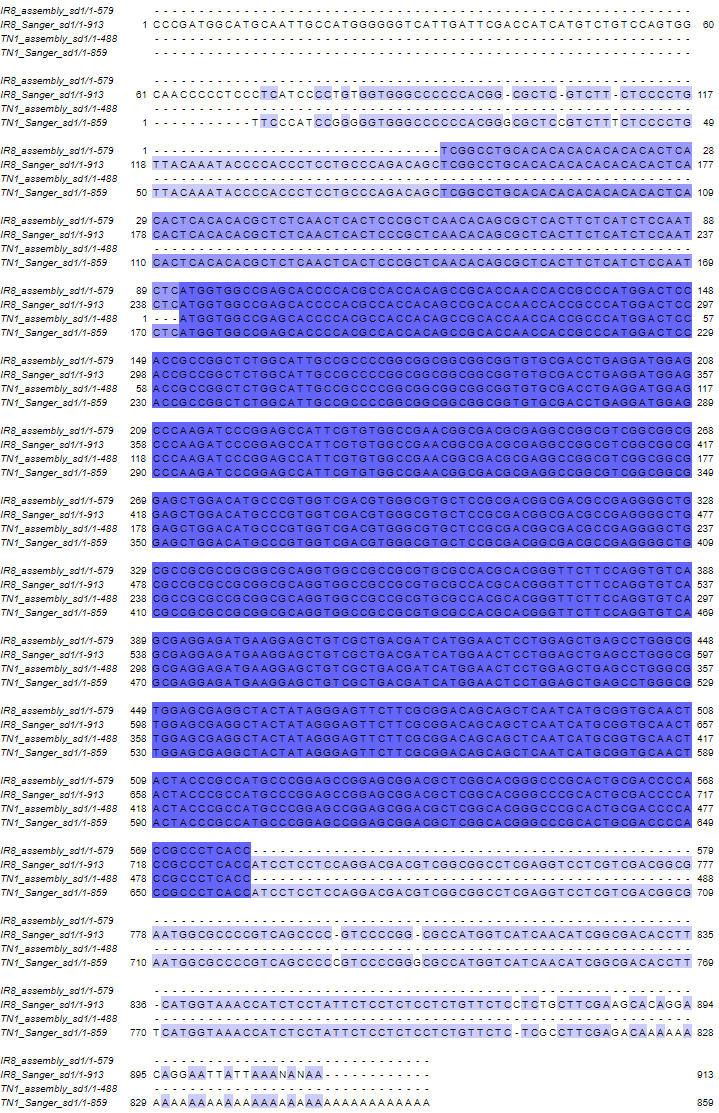


**Figure S2.** **Alignment of *sd*1 of TN1 (chr 1:40,361,934-40,362,421), IR8 (chr 1: 39,824,196-39,824,774) vs. their Sanger sequencing.** Those highlighted in blue are 100% identical.


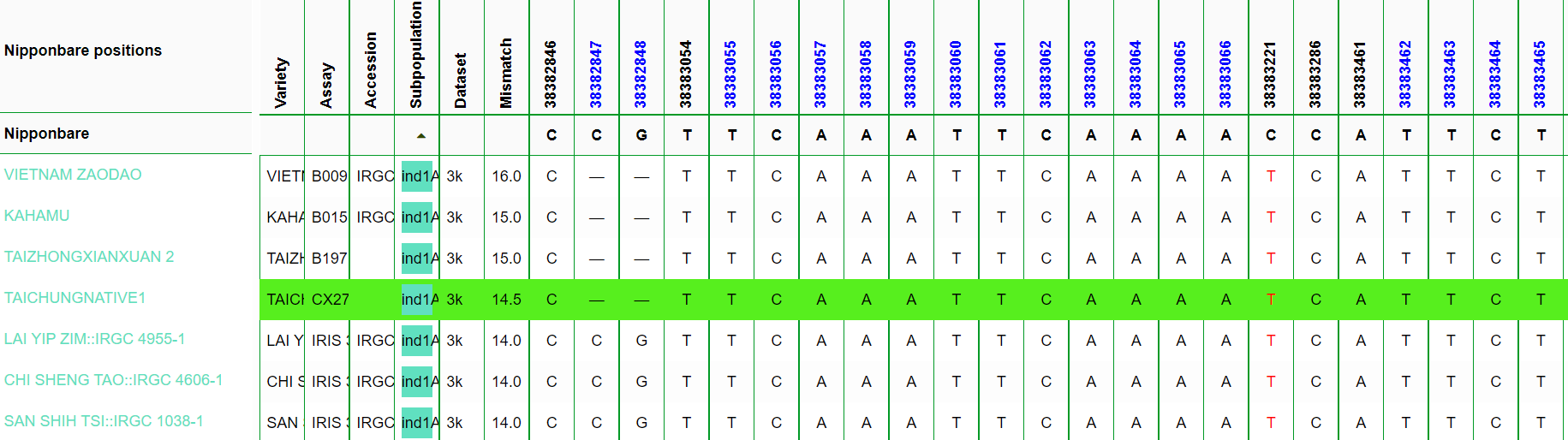


**Figure S3**. **Screenshot of the Genotype search result in the SNP-Seek database for TAICHUNGNATIVE1 (CX270).**


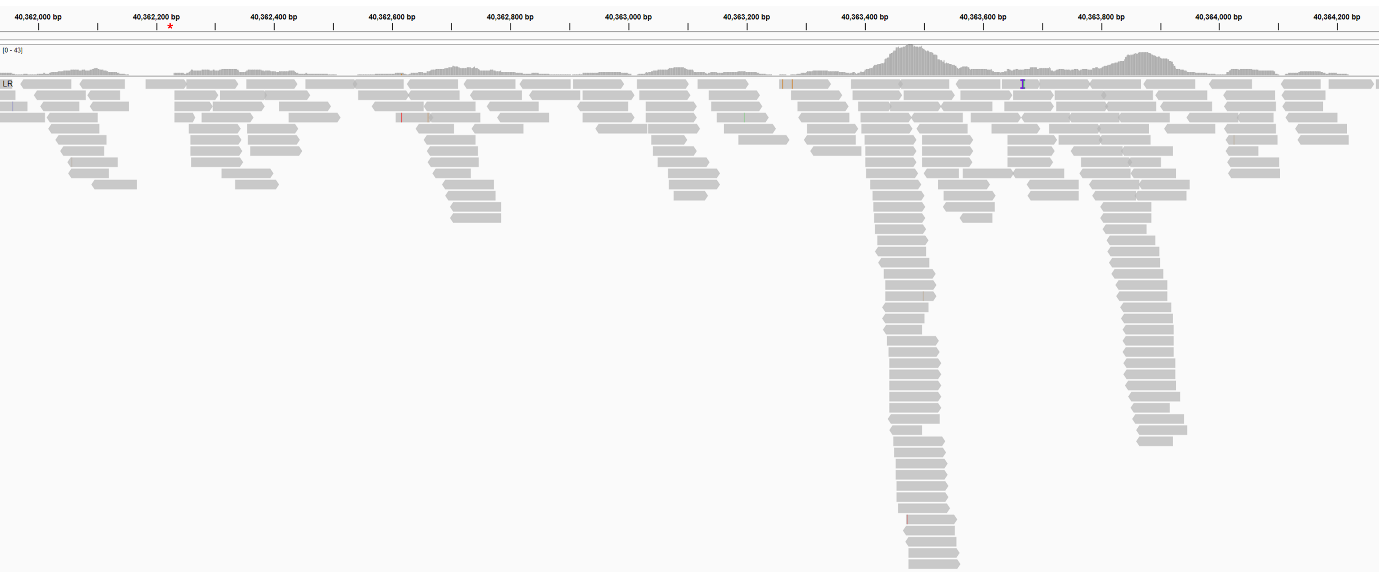


**Figure S4**. **Mapping of TAICHUNGNATIVE1 (CX270) properly paired reads in the TN1 sd1 gene region.** The red asterisk marks the location of the nucleotide that caused the 382-bp deletion in the sd1 gene of TN1 and IR8, instead of 383-bp.


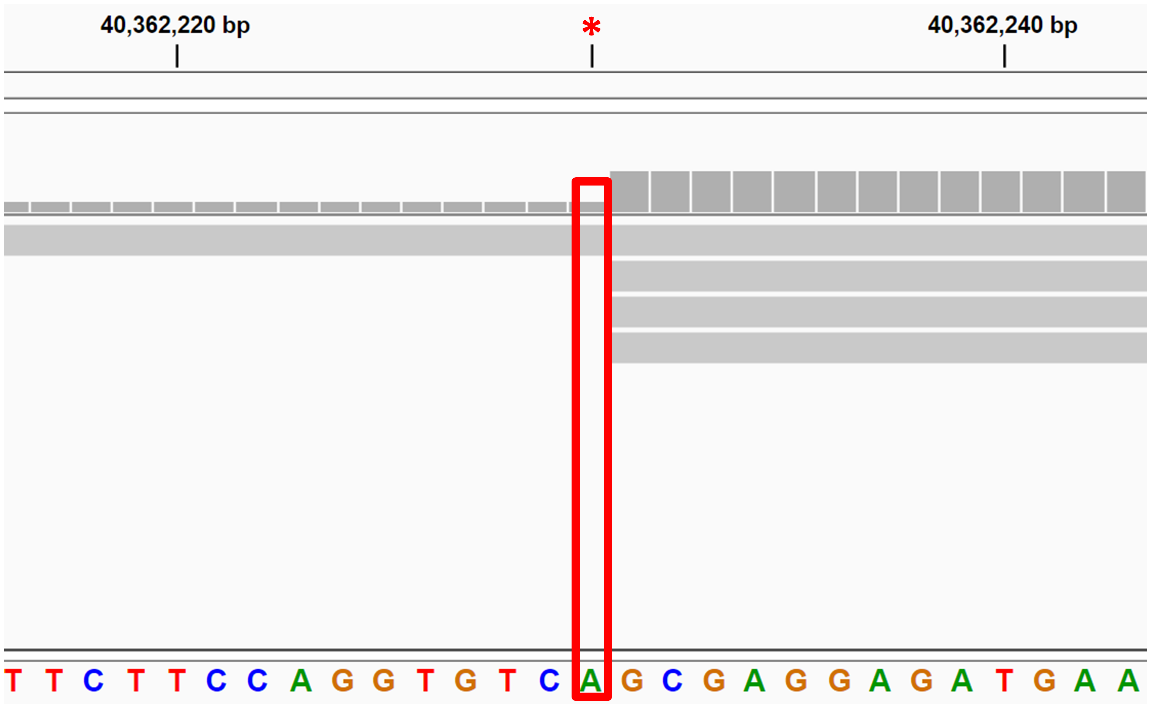


**Figure S5. Magnified view of the mapping of TAICHUNGNATIVE1 (CX270) reads at TN1 chromosome 1 position 40,362,230**. The letters are the nucleotides of TN1 chromosome 1. The red box indicates the region of interest. The vertical bars are mapping coverage while the horizontal bars are the reads. The gray color of the reads means that their sequence is similar to the TN1 reference genome.


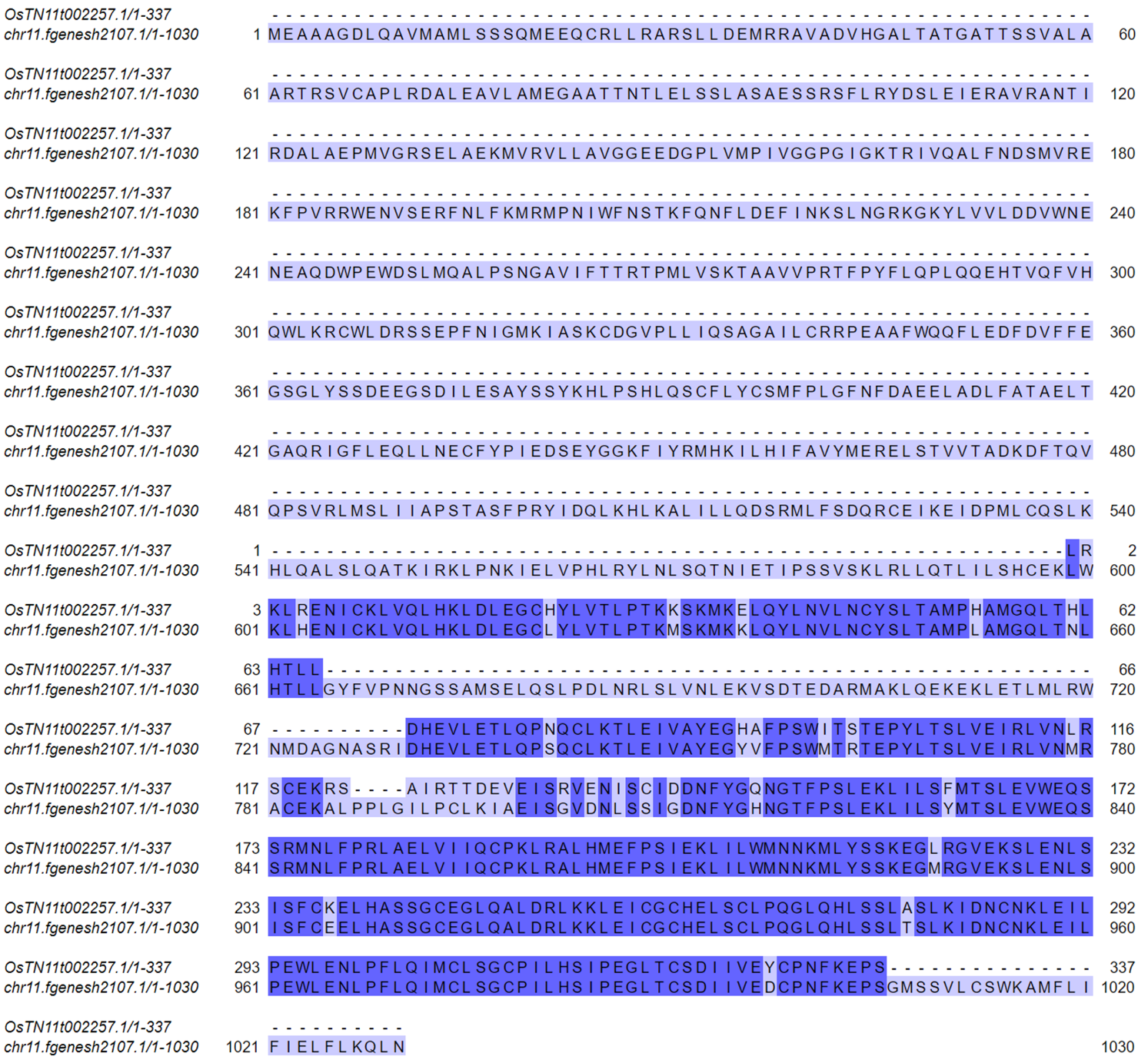


**Figure S6**. **Alignment of TN1 OsTN11t002257.1 against Tetep Pi54 (chr11.fgenesh2107.1).** Those highlighted in blue are 100% identical.


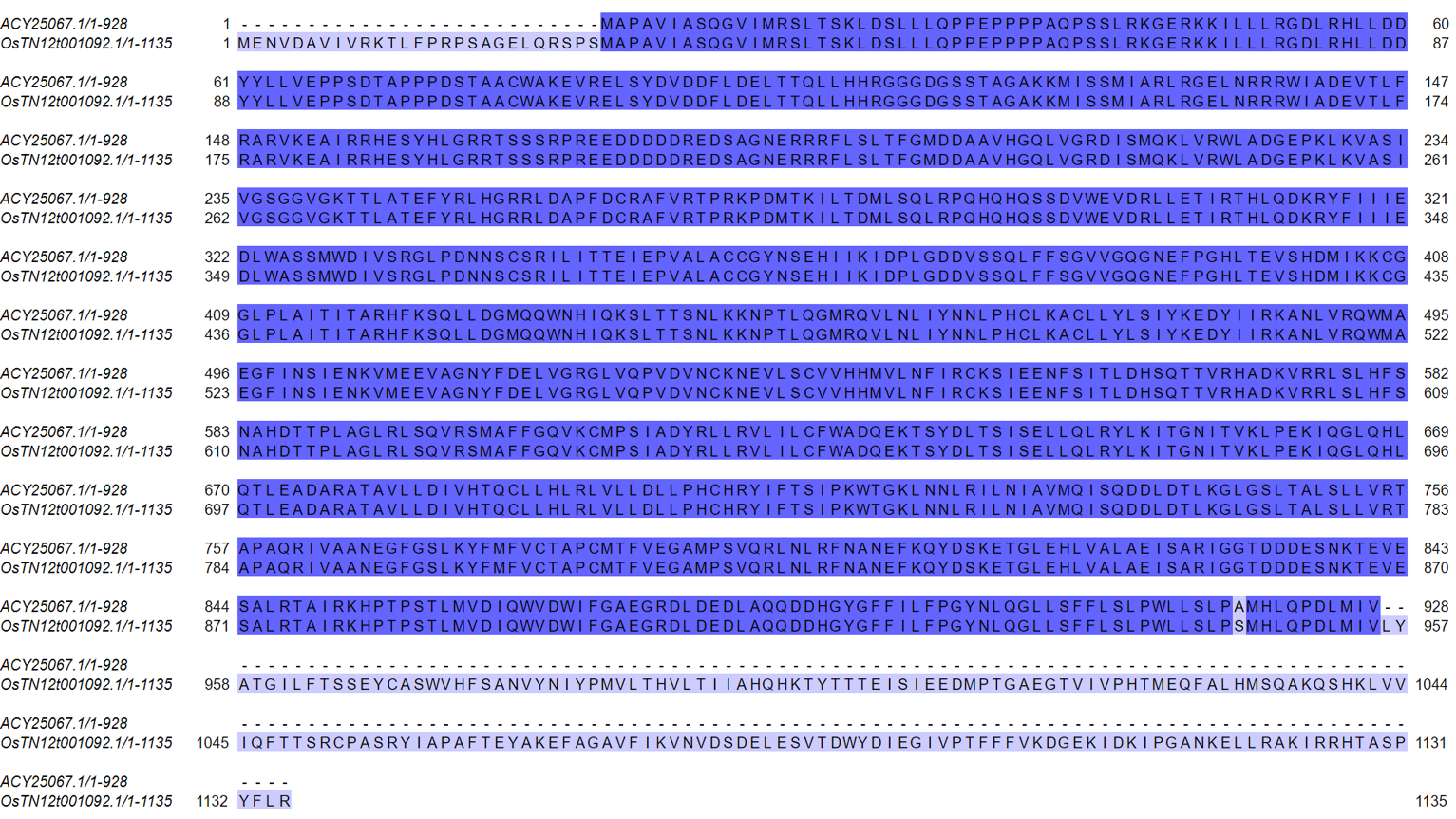


**Figure S7.** **Alignment of Pi-ta protein sequence of TN1 (OsTN12t001092.1) against Yashiro-mochi (ACY25067.1).** Those highlighted in blue are 100% identical.


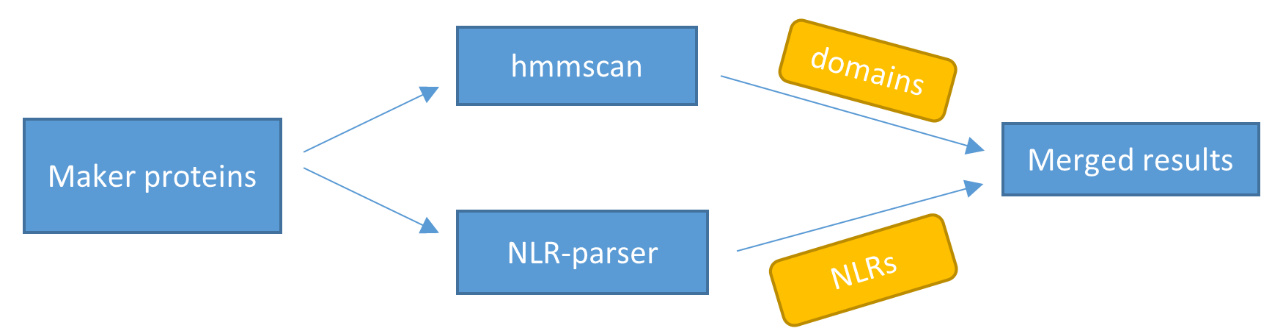


**Figure S8. R gene prediction workflow.** The merged data from hmmscan and NLR-parser represent the official results for the R gene search.

**
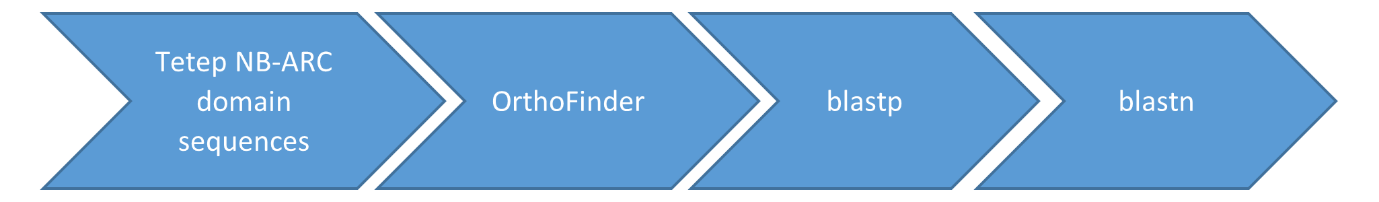
**

**Figure S9. Schematic diagram showing the Tetep R-gene search in the TN1 genome.** The inputs to OrthoFinder were protein sequences. For blastp, Tetep NB-ARC domains were searched against the TN1 proteins, while for blastn the Tetep NB-ARC coding sequences were aligned against the TN1 genome.


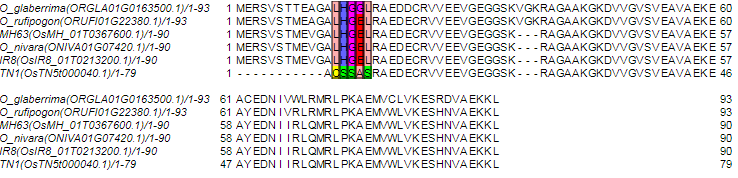


**Figure S10a. OsTN5g000040.1 (hypothetical protein) translated CDS alignment.** The TN1 PS sites and their probability of being under PS: Cys2 (99.45%), Ser3 (97.81%), Ser4 (98.05%), Ala5 (79.83%), and Ser6 (96.57%).

**
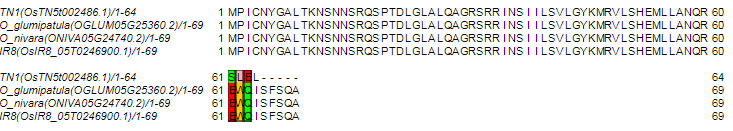
**

**Figure S10b. OsTN5g002486.1 translated CDS alignment.** The TN1 PS sites and their probability of being under PS: Ser61 (99.95%), Leu62 (99.01%), and Glu63 (99.02%).

**
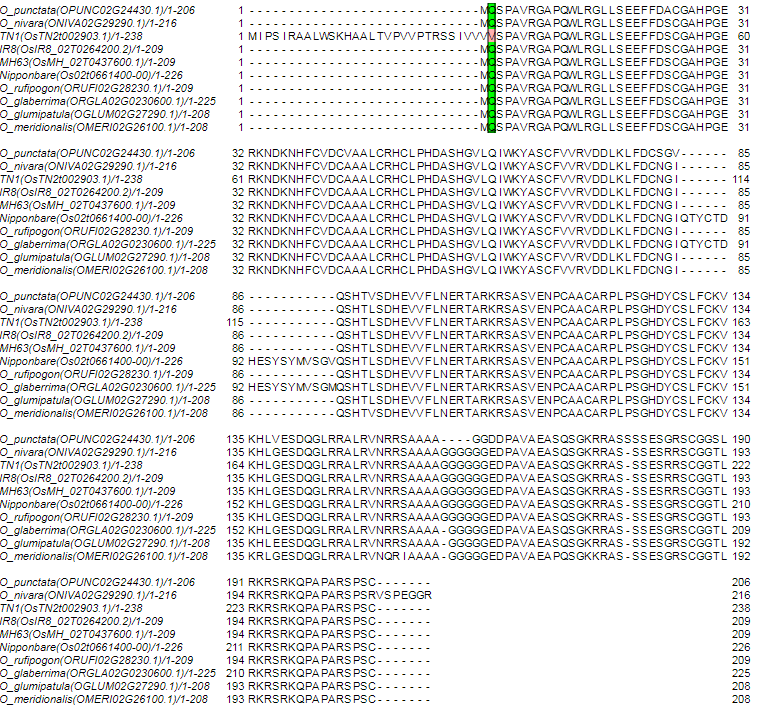
**

**Figure S10c. OsTN2g002903.1 (PLATZ transcription factor family protein) translated CDS alignment.** The TN1 PS site and the probability of being under PS: Val31 (99.33%).

**
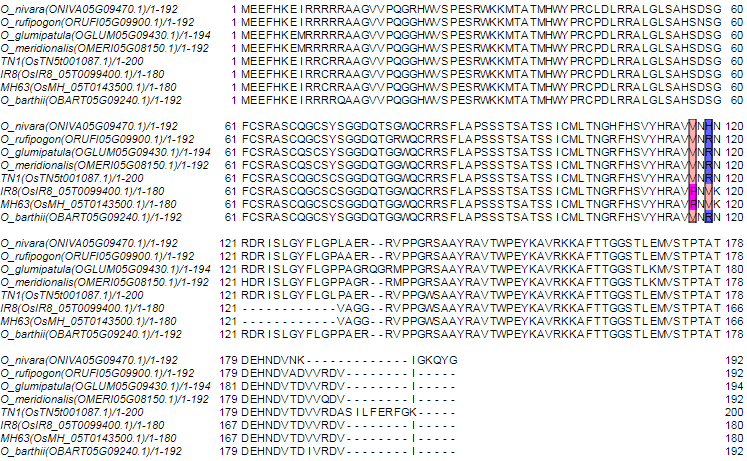
**

**Figure S10d. OsTN5g001087.1 (GA 3β-hydroxylase) translated CDS alignment.** The TN1 PS sites and their probability of being under PS: Val117 (81.50%) and Arg119 (81.61%).

**
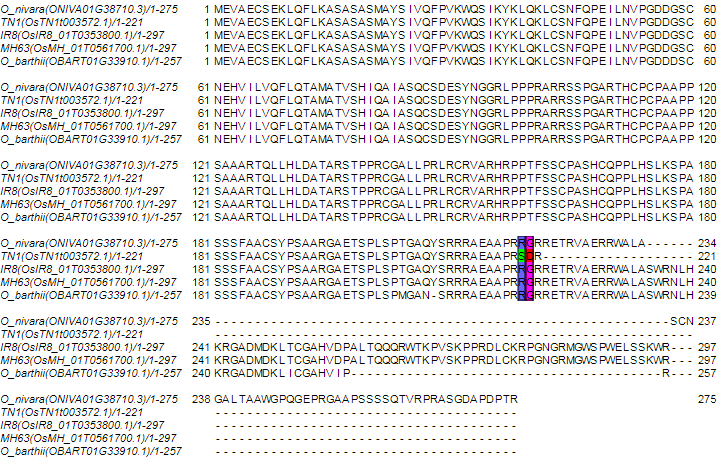
**

**Figure S10e. OsTN1g003572.1 (armadillo/beta-catenin repeat protein-like) translated CDS alignment.** The TN1 PS sites and their probability of being under PS: Ser219 (85.68%) and Asp220 (79.28%).

**
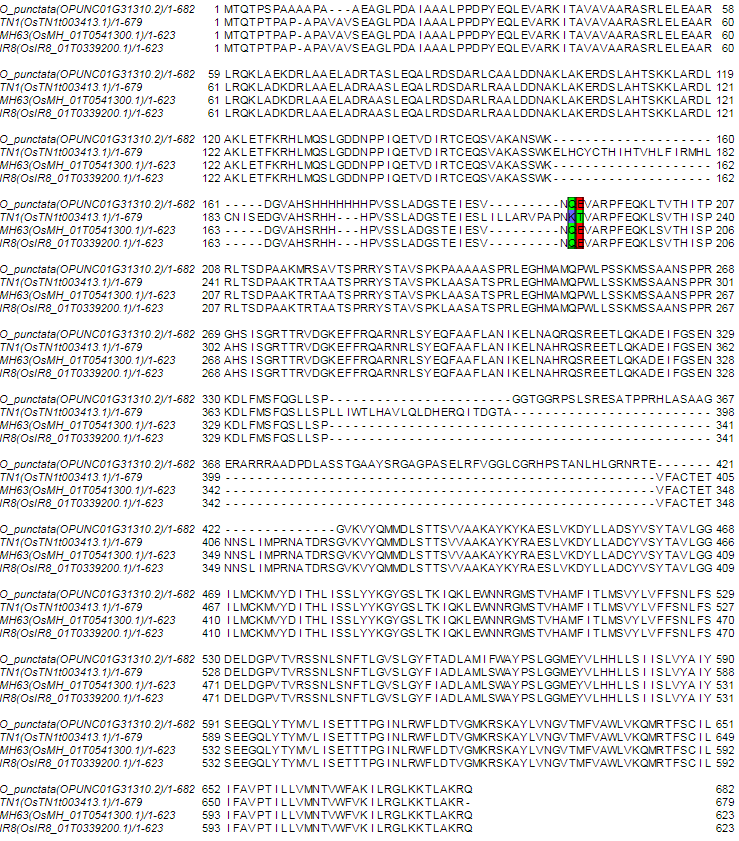
**

**Figure S10f. OsTN1g003413.1 (transmembrane protein 56 isoform X1) translated CDS alignment.** The TN1 PS sites and their probability of being under PS: Lys223 (80.52%) and Thr224 (96.54%).

**
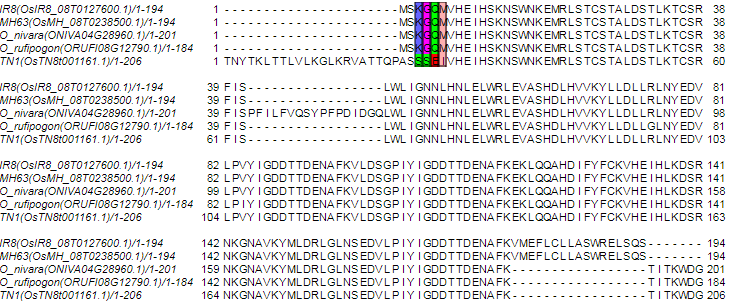
**

**Figure S10g. OsTN8g001161.1 (probable TPP C) translated CDS alignment.** The TN1 PS sites and their probability of being under PS: Ser25 (99.59%), Ser26 (98.79%), Glu27 (95.75%), and Ile28 (95.74%).

**
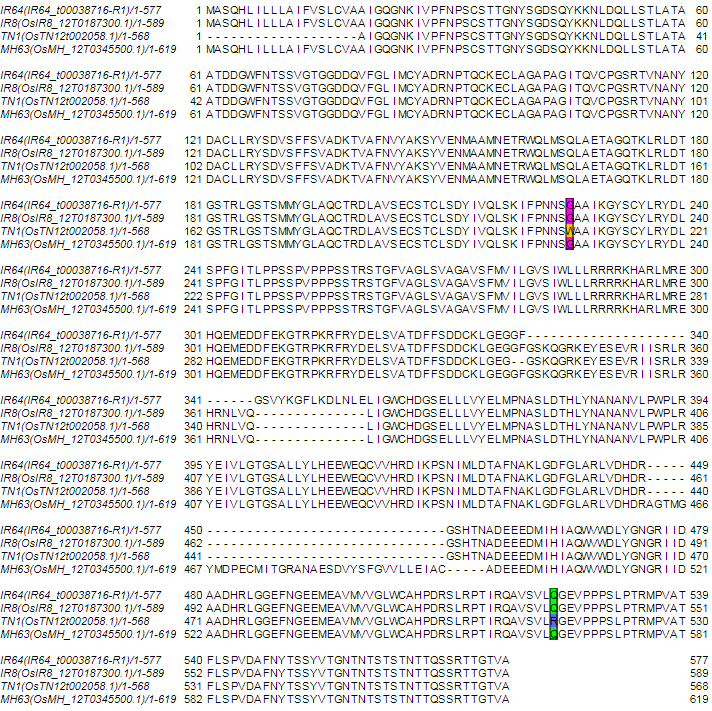
**

**Figure S10h. OsTN12g002058.1 (L-type lectin-domain containing receptor kinase IX.1-like) translated CDS alignment.** The TN1 PS sites and their probability of being under PS: Trp207 (63.59%) and Arg514 (43.54%).

**
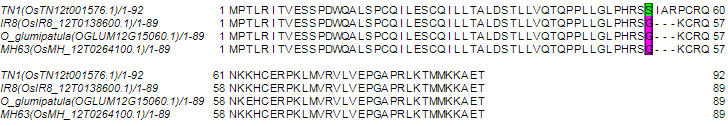
**

**Figure S10i. OsTN12g001576.1 translated CDS alignment.** The TN1 PS site and the probability of being under PS: Ser53 (85.69%).

**
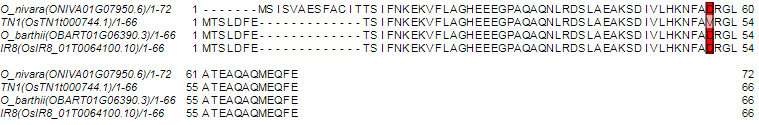
**

**Figure S10j. OsTN1g000744.1 (KARI, chloroplastic) translated CDS alignment.** The TN1 PS site and the probability of being under PS: Val51 (90.33%).

**
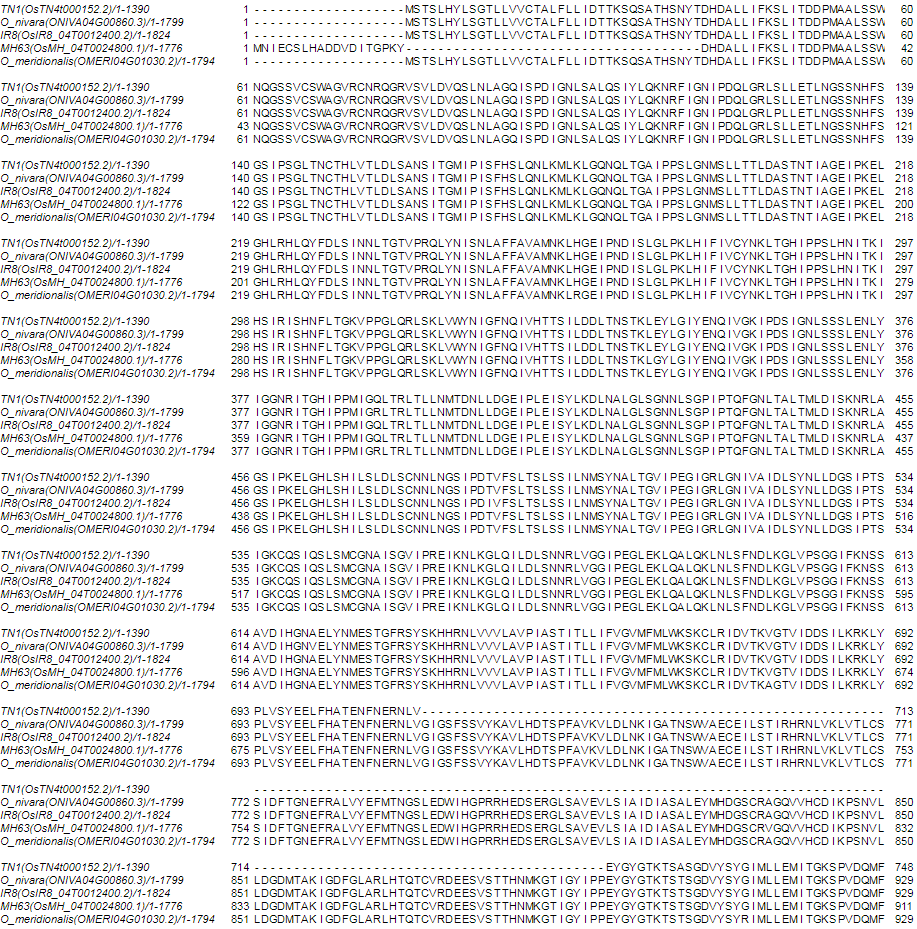
**

**Figure S10k. OsTN4g000152.2 (probable LRR receptor-like serine/threonine-protein kinase At3g47570).** The TN1 PS sites and their probability of being under PS: Lys1182 (69.84%) and Thr1286 (60.54%).

**
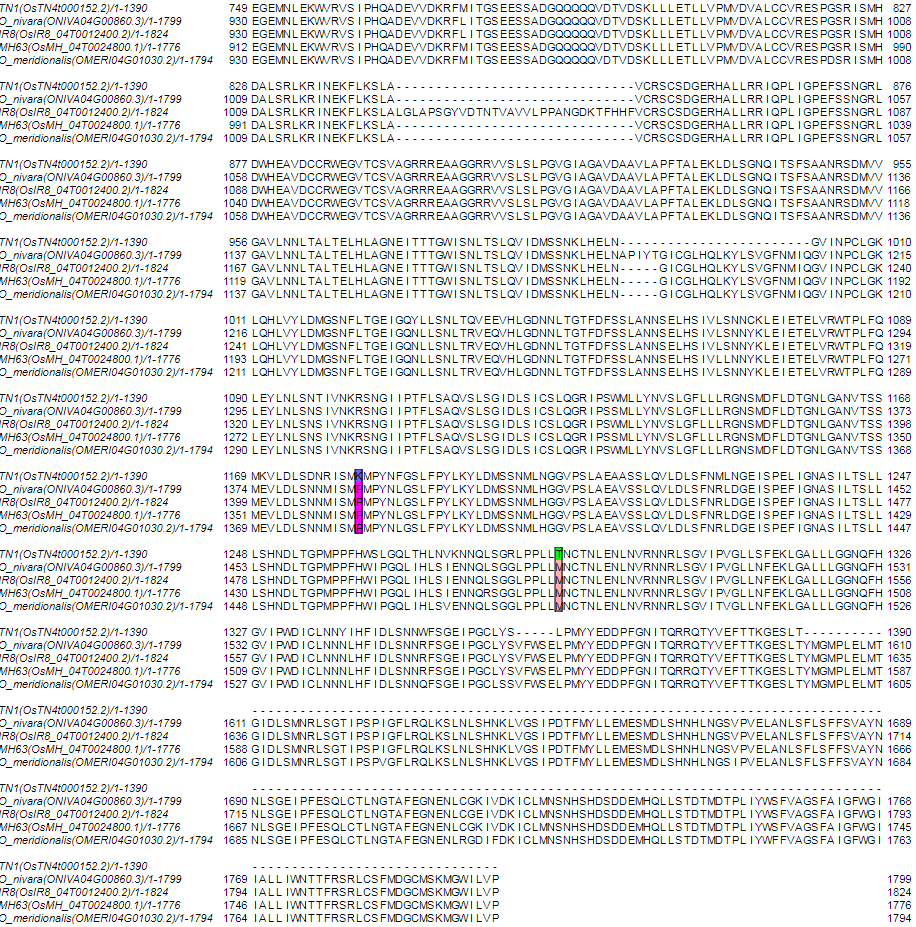
**

**Figure S10k continued.**

1. **SUPPLEMENTARY TABLES**

**Table S1. Number of Tetep NLRs that are found in TN1, MH63, R498 and Nipponbare.** The sum of the NLRS with orthologues and with blastp hits ≥ 50%, (429 for TN1, 423 for MH63, 436 for R498, 438 for Nipponbare), did not differ much.

| **Tetep (455 NLRs)** | | **TN1** | **MH63** | **R498** | **Nipponbare** |
| --- | --- | --- | --- | --- | --- |
| NLRs with orthologues(defined by OrthoFinder) | | 322 | 335 | 332 | 360 |
| NLRs without orthologues | Blastp Identity ≥ 50% | 107 | 88 | 104 | 78 |
|  | Blastp Identity < 50% | 26 | 32 | 19 | 17 |

**Table S2: Proportion of R genes that have been tested either in TP309 or Shin2.** The numbers on this table were used in the Chi-square computation in R.

|  | **Tested** | **Resistant (either TP309 or Shin2)** |
| --- | --- | --- |
| **Tetep NBS** | 219 | 90 |
| **Orthologs in TN1(defined by OrthoFinder)** | 170 | 69 |

**Table S3. Gene Ontology (GO) terms of the TN1 genes under positive selection.** The GOs on this table were used as input in REVIGO.

| Gene name | protein name | GO id | meaning |
| --- | --- | --- | --- |
| OsTN5g000040 | hypothetical protein | - | - |
| OsTN5g002486 | - | - | - |
| OsTN2g002903 | PLATZ transcription factor family protein | - | - |
| OsTN5g001087 | GA 3beta-hydroxylase | Biological Process |  |
|  |  | GO:0009416 | response to light stimulus |
|  |  | GO:0009686 | gibberellin biosynthetic process |
|  |  | GO:0009826 | unidimensional cell growth |
|  |  | GO:0009908 | flower development |
|  |  | GO:0055114 | oxidation-reduction process |
|  |  | Molecular Function |  |
|  |  | GO:0016707 | gibberellin 3-beta-dioxygenase activity |
|  |  | GO:0045544 | gibberellin 20-oxidase activity |
| OsTN1g003572 | armadillo/beta-catenin repeat protein-like | - | - |
| OsTN1g003413 | transmembrane protein 56 isoform X1 | Biological Process |  |
|  |  | GO:0055085 | transmembrane transport |
|  |  | Cellular Component |  |
|  |  | GO:0005886 | plasma membrane |
|  |  | GO:0016021 | integral component of membrane |
|  |  | Molecular Function |  |
|  |  | GO:0022857 | transmembrane transporter activity |
| OsTN8g001161 | probable trehalose-phosphate phosphatase C | Biological Process |  |
|  |  | GO:0005992 | trehalose biosynthetic process |
|  |  | GO:0016311 | dephosphorylation |
|  |  | Molecular Function |  |
|  |  | GO:0004805 | trehalose-phosphatase activity |
| OsTN12g002058 | L-type lectin-domain containing receptor kinase IX.1-like | Biological Process |  |
|  |  | GO:0002229 | defense response to oomycetes |
|  |  | GO:0006468 | protein phosphorylation |
|  |  | GO:0042742 | defense response to bacterium |
|  |  | Cellular Component |  |
|  |  | GO:0016021 | integral component of membrane |
|  |  | GO:0005886 | plasma membrane |
|  |  | Molecular Function |  |
|  |  | GO:0004675 | transmembrane receptor protein serine/threonine kinase activity |
|  |  | GO:0005524 | ATP binding |
| OsTN12g001576 | - | - | - |
| OsTN1g000744 | Ketol-acid reductoisomerase, chloroplastic | Biological Process |  |
|  |  | GO:0009097 | isoleucine biosynthetic process |
|  |  | GO:0009099 | valine biosynthetic process |
|  |  | GO:0055114 | oxidation-reduction process |
|  |  | Cellular Component |  |
|  |  | GO:0005739 | mitochondrion |
|  |  | GO:0009507 | chloroplast |
|  |  | Molecular Function |  |
|  |  | GO:0046872 | metal ion binding |
|  |  | GO:0004455 | ketol-acid reductoisomerase activity |
| OsTN4g000152 | probable LRR receptor-like serine/threonine-protein kinase At3g47570 | Biological Process |  |
|  |  | GO:0006468 | protein phosphorylation |
|  |  | GO:0009755 | hormone-mediated signaling pathway |
|  |  | Cellular Component |  |
|  |  | GO:0005886 | plasma membrane |
|  |  | GO:0016021 | integral component of membrane |
|  |  | Molecular Function |  |
|  |  | GO:0004674 | protein serine/threonine kinase activity |
|  |  | GO:0005515 | protein binding |
|  |  | GO:0005524 | ATP binding |

**Table S4. Statistics of coding sequences used in the PosiGene run and their source link.** With the exception of TN1, IR64 and MH63, all the coding sequences of the input species were downloaded from the Gramene ftp website. Because the ftp link can change without prior notice, users wanting to download the same data should refer to release 62 and oge release 3, if ever the links had changed.

| **organism** | **Version/**  **Date downloaded or extracted** | **# CDS** | **Total length of coding sequences** | **source link** |
| --- | --- | --- | --- | --- |
| TN1 | April 26, 2020 | 37,952 | 45,684,501 | - |
| IR8 | May 10, 2020 | 56,823 | 76,985,769 | <http://ftp.gramene.org/oge/release-3/fasta/oryza_indicair8/dna/> |
| IR64 | May 10, 2020 | 41,458 | 45,637,530 | <https://rootomics.dna.affrc.go.jp/en/research/IR64> |
| MH63 | MH63RS2 | 83,258 | 109,797,326 | <http://rice.hzau.edu.cn/cgi-bin/rice_rs2/download_ext> |
| *Oryza nivara* | v1.0 | 48,360 | 61,006,575 | [http://ftp.gramene.org/archives/PAST_RELEASES/release-62/fasta/oryza_nivara/cds/](http://ftp.gramene.org/archives/PAST_RELEASES/release-62/fasta/oryza_nivara) |
| *Oryza rufipogon* | May 10, 2020 | 50,219 | 59,474,618 | <http://ftp.gramene.org/oge/release-3/fasta/oryza_rufipogon/dna/> |
| 9311 | ASM465v1 | 40,745 | 45,520,686 | [http://ftp.gramene.org/archives/PAST_RELEASES/release-62/fasta/oryza_indica/cds/](http://ftp.gramene.org/archives/PAST_RELEASES/release-62/fasta/oryza_indica/) |
| Nipponbare | March 24, 2020 | 42,313 | 42,136,737 | <https://rapdb.dna.affrc.go.jp/download/irgsp1.html> |
| *Oryza barthii* | v1 | 41,595 | 50,568,141 | [http://ftp.gramene.org/archives/PAST_RELEASES/release-62/fasta/oryza_barthii/cds/](http://ftp.gramene.org/archives/PAST_RELEASES/release-62/fasta/oryza_barthii/) |
| *Oryza brachyantha* | v1.4b | 32,037 | 34,004,031 | [http://ftp.gramene.org/archives/PAST_RELEASES/release-62/fasta/oryza_brachyantha/cds/](http://ftp.gramene.org/archives/PAST_RELEASES/release-62/fasta/oryza_brachyantha/) |
| *Oryza glaberrima* | V1 | 33,164 | 36,293,721 | [http://ftp.gramene.org/archives/PAST_RELEASES/release-62/fasta/oryza_glaberrima/cds/](http://ftp.gramene.org/archives/PAST_RELEASES/release-62/fasta/oryza_glaberrima/) |
| *Oryza glumipatula* | v1.5 | 46,893 | 58,081,746 | [http://ftp.gramene.org/archives/PAST_RELEASES/release-62/fasta/oryza_glumipatula/cds/](http://ftp.gramene.org/archives/PAST_RELEASES/release-62/fasta/oryza_glumipatula/) |
| *Oryza punctata* | v1.2 | 41,060 | 53,482,920 | [http://ftp.gramene.org/archives/PAST_RELEASES/release-62/fasta/oryza_punctata/cds/](http://ftp.gramene.org/archives/PAST_RELEASES/release-62/fasta/oryza_punctata/) |
| *Oryza meridionalis* | v1.3 | 43,455 | 55,214,595 | [http://ftp.gramene.org/archives/PAST_RELEASES/release-62/fasta/oryza_meridionalis/cds/](http://ftp.gramene.org/archives/PAST_RELEASES/release-62/fasta/oryza_meridionalis/) |
| *Oryza longistaminata* | v1.0 | 31,686 | 34,731,219 | [http://ftp.gramene.org/archives/PAST_RELEASES/release-62/fasta/oryza_longistaminata/cds/](http://ftp.gramene.org/archives/PAST_RELEASES/release-62/fasta/oryza_longistaminata/) |
| *Brachypodium distachyon* | v3.0 | 52,972 | 66,747,974 | [http://ftp.gramene.org/archives/PAST_RELEASES/release-62/fasta/brachypodium_distachyon/cds](http://ftp.gramene.org/archives/PAST_RELEASES/release-62/fasta/brachypodium_distachyon)/ |
| *Eragrostis tef* | ASM97063v1 | 41,555 | 48,850,019 | [http://ftp.gramene.org/archives/PAST_RELEASES/release-62/fasta/eragrostis_tef/cds/](http://ftp.gramene.org/archives/PAST_RELEASES/release-62/fasta/eragrostis_tef) |
| *Leersia perrieri* | V1.4 | 38,960 | 52,135,503 | [http://ftp.gramene.org/archives/PAST_RELEASES/release-62/fasta/leersia_perrieri/cds/](http://ftp.gramene.org/archives/PAST_RELEASES/release-62/fasta/leersia_perrieri) |
| *Panicum hallii fil2* | v3.1 | 44,192 | 54,354,546 | [http://ftp.gramene.org/archives/PAST_RELEASES/release-62/fasta/panicum_hallii_fil2/cds/](http://ftp.gramene.org/archives/PAST_RELEASES/release-62/fasta/panicum_hallii_fil2) |
| *Panicum hallii hal2* | v2.1 | 42,523 | 51,996,270 | <http://ftp.gramene.org/archives/PAST_RELEASES/release-62/fasta/panicum_hallii_hal2/cds/> |
| *Setaria italica* | v2.0 | 41,023 | 45,897,567 | <http://ftp.gramene.org/archives/PAST_RELEASES/release-62/fasta/setaria_italica/cds/> |
| *Sorghum bicolor* | NCBIv3 | 47,110 | 57,859,353 | <http://ftp.gramene.org/archives/PAST_RELEASES/release-62/fasta/sorghum_bicolor/cds/> |
| *Triticum aestivum* | IWGSC | 133,346 | 177,672,347 | <http://ftp.gramene.org/archives/PAST_RELEASES/release-62/fasta/triticum_aestivum/cds/> |
| *Zea mays* | v4 | 131,585 | 190,385,016 | [http://ftp.gramene.org/archives/PAST_RELEASES/release-62/fasta/zea_mays/cds/](http://ftp.gramene.org/archives/PAST_RELEASES/release-62/fasta/zea_mays) |

**Dataset S1:** Predicted R genes in TN1, their Pfam domains, NLR-Parser result and R gene classification

**Dataset S2:** Results of finding Tetep NLRs in TN1

**Dataset S3:** Blastp and tblastn hits of the cloned R genes to the TN1 and Tetep genome

**Dataset S4:** Haplotype and variety order of Pi54 and Pi-ta from SNP-Seek

**Dataset S5:** SNP effect results from SNP-Seek
